# Time-Course Comparative Metabolite Profiling under Osmotic Stress in Tolerant and Sensitive Tibetan Hulless Barley

**DOI:** 10.1155/2018/9415409

**Published:** 2018-12-23

**Authors:** Hongjun Yuan, Xingquan Zeng, Jian Shi, Qijun Xu, Yulin Wang, Dunzhu Jabu, Zha Sang, Tashi Nyima

**Affiliations:** ^1^State Key Laboratory of Barley and Yak Germplasm Resources and Genetic Improvement, Lhasa 850002, China; ^2^Institute of Agricultural Research, Tibet Academy of Agricultural and Animal Husbandry Sciences, Lhasa 850002, China; ^3^Wuhan Metware Biotechnology Co., Ltd, Wuhan 430070, China; ^4^Tibet Academy of Agricultural and Animal Husbandry Sciences, Lhasa, Tibet 850002, China

## Abstract

Tibetan hulless barley is widely grown in the extreme environmental conditions of the Qinghai-Tibet Plateau which is characterized by cold, high salinity, and drought. Osmotic stress always occurs simultaneously with drought and its tolerance is a vital part of drought tolerance. The diversity of metabolites leading to osmotic stress tolerance was characterized using widely-targeted metabolomics in tolerant (XL) and sensitive (D) accessions submitted to polyethylene glycol. XL regulated a more diverse set of metabolites than D, which may promote the establishment of a robust system to cope with the stress in XL. Compounds belonging to the group of flavonoids, amino acids, and glycerophospholipids constitute the core metabolome responsive to the stress, despite the tolerance levels. Moreover, 8 h appeared to be a critical time point for stress endurance involving a high accumulation of key metabolites from the class of nucleotide and its derivative which provide the ultimate energy source for the synthesis of functional carbohydrates, lipids, peptides, and secondary metabolites in XL. This intrinsic metabolic adjustment helped XL to efficiently alleviate the stress at the later stages. A total of 22 diverse compounds were constantly and exclusively regulated in XL, representing novel stress tolerance biomarkers which may help improving stress tolerance, especially drought, in hulless barley.

## 1. Introduction

Barley ranks as the fourth most widely produced cereal in the world and is one of the most economically important crops [1]. Basically, it is classified into two main categories according to the grain type: hulled and hulless barley. Hulless barley (*Hordeum vulgare *L. var.* nudum *Hook. f.) differs from the hulled barley by the loose husk cover of caryopses that makes the grain hull-free at maturity [2]. Recently, hulless barley has attracted increasing attention due to its health-promoting effects (high *β*-glucan content, low amylose content, etc.), high-feeding value, and high-malt quality [3]. The crop is widely grown in the world but is more significant in East Asian countries such as China, especially in Tibet [4].

Hulless barley is the major staple food crop for Tibetans, occupying ~70% of the crop lands on the Qinghai-Tibet Plateau in China [5]. However, because the crop is grown in the high stressful conditions of the Qinghai-Tibet Plateau, the yield and productivity are significantly impaired by abiotic stresses such as cold, salinity, and drought [6]. Therefore, studying abiotic stress adaptation in hulless barley, especially drought tolerance mechanisms, is of paramount importance.

Drought is the single most critical threat to world food security and with the limited water supply in the world, future food demand for rapidly growing populations will further aggravate the effects of drought [7]. Although plants are sessile organisms, they have developed a variety of mechanisms in order to adapt to any stress condition including drought. Drought stress affects physiological, biochemical, and molecular processes, such as photosynthesis, respiration, translocation, ion uptake, metabolism, and growth promoters [8]. In hulless barley, integrative functional genomics approach was employed to elucidate the molecular basis of drought tolerance. The genes* HbSINA4* and* HbSYR1* were cloned and functionally validated as important genes for drought tolerance and water retention [9, 10]. Based on transcriptome analysis, 853 differentially expressed genes were detected as involved in drought response and categorized into nine clusters enriched in various biological pathways in a drought tolerant hulless barley genotype [11]. More recently, by comparing two genotypes with contrasting tolerance to drought, Liang et al. [12] reported that the tolerant genotype has more upregulated genes than the sensitive one and found several exclusively enriched pathways in the tolerant genotype. Overall, these studies provided important insights into the molecular mechanisms of hulless barley tolerance to drought.

In contrast to the genomic studies, no omics-scale study of metabolites active under drought has been performed in hulless barley, although the production and abundance of several key compatible metabolites have been proven to be critical for plant drought tolerance [13]. Metabolite profiling contributes to the understanding of plants' stress responses through the detection and robust quantification of active compounds including organic acids, sugars, sugar alcohols, amino acids, and secondary metabolites [14]. Extensive studies have been performed in various plant species to understand the plant's responses to drought stress at the metabolic level [15–18].

Osmotic stress always occurs simultaneously with drought and its tolerance is a vital part of drought tolerance [13]. Given that finely regulating soil moisture under controlled conditions is notoriously difficult, hydroponic osmotic stress induced by polyethylene glycol (PEG) was used in this study. To provide a comprehensive understanding of the role of the metabolites that form the basis of osmotic stress tolerance in a special crop species that has evolved for millennia in a drought-prone environment, the metabolomes of two Tibetan hulless barley accessions differing in their tolerance to PEG-simulated drought stress were compared at five time points under stress using the widely targeted metabolomics platform.

## 2. Materials and Methods

### 2.1. Plant Materials

Two hulless barley accessions (XL and D) were used in this study. They were selected from 1,700 germplasm resources, initially screened for osmotic stress tolerance induced by polyethylene glycol (PEG) 6000. XL displays a strong tolerance while D is highly sensitive to osmotic stress.

### 2.2. Plant Growth and Stress Treatment

Seeds of the two accessions were sown in a plastic tray (37 × 35 × 25 cm) filled with nutritional soil: vermiculite (1:1). They were grown in a plant incubator set at 25°C, 2000 *μ*mol.m^−2^.s^−1^. At the 2-3 leaf stage, seedlings were removed from the tray and thoroughly washed with tap water. Uniform seedlings were then transferred into *½*  strength Hoagland nutrient solution [19]. After seven days of growth, half of the plants were transferred into a new medium supplemented with 21% PEG6000. PEG has been effectively used to simulate osmotic stress caused by drought with limited metabolic interferences because it is less likely to be absorbed by plants and is not phytotoxic [20]. Fresh leaves were sampled early in the morning from individual plants at 0, 0.5, 1, 2, 4, 8, 12, 24, and 48 h from control treatment (CK) and stress treatment (S). For each accession at a given time point and treatment, three biological replicates from three different plants were collected. The materials were harvested from the youngest fully expanded leaves and were immediately snap-frozen in liquid nitrogen. The frozen leaf samples were afterwards ground in liquid nitrogen, stored in 15 mL Falcon tubes at −80°C, and later used for biochemical measurements and metabolomic analyses.

### 2.3. Leaf Malondialdehyde Content Measurement

The amount of malondialdehyde (MDA) in the leaf samples was measured as thiobarbituric acid-reactive material from centrifuged leaf extracts in 10% trichloroacetic acid [21]. The value for nonspecific absorption at 600 nm was subtracted from the 532 nm reading. The concentration of MDA was calculated using its extinction coefficient of 155 mM^−1^.cm^−1^ and expressed as nmol.g^−1^.FW. Measurements were performed in triplicate at nine time points as mentioned above.

### 2.4. Metabolite Profiling

#### 2.4.1. Sample Preparation and Extraction

The leaf samples collected at 0, 1, 4, 8, 24, and 48 h were crushed using a mixer mill (MM 400, Retsch) with a zirconia bead for 1.5 min at 30 Hz. One hundred (100) mg powder was weighed and aliquots were extracted overnight at 4°C with 1 ml 70% aqueous methanol. Following centrifugation at 10,000 g for 10 min, the extracts were absorbed (CNWBOND Carbon-GCB SPE Cartridge, 250 mg, 3 ml; ANPEL, Shanghai, China, http://www.anpel.com.cn/Search.aspx?Types=6&Type=0&KeyWord=CnwBOND) and filtrated (SCAA-104, 0.22 *μ*m pore size; ANPEL, Shanghai, China) before the LC-MS analysis [22].

#### 2.4.2. HPLC Conditions

The sample extracts were analyzed using an LC-ESI-MS/MS system (HPLC, Shim-pack UFLC SHIMADZU CBM30A system, https://www.shimadzu.com.cn; MS, Applied Biosystems 6500 Q TRAP, https://www.appliedbiosystems.com.cn). The analytical conditions were as follows, HPLC: column, Waters ACQUITY UPLC HSS T3 C18 (1.8 *μ*m, 2.1 mm×100 mm); solvent system, water (0.04% acetic acid): acetonitrile (0.04% acetic acid); gradient program, 100:0 V/V at 0 min, 5:95 V/V at 11 min, 5:95 V/V at 12 min, 95:5 V/V at 12.1 min, 95:5 V/V at 15 min; flow rate, 0.40 ml.min^−1^; temperature, 40°C; injection volume: 2 *μ*l. The effluent was alternatively connected to an ESI-triple quadrupole-linear ion trap (Q TRAP)-MS.

#### 2.4.3. ESI-Q TRAP-MS/MS

Linear ion trap (LIT) and triple quadrupole (QQQ) scans were acquired on a Q TRAP-MS, API 6500 Q TRAP LC/MS/MS System, equipped with an ESI Turbo Ion-Spray interface, operating in a positive ion mode and controlled by the Analyst 1.6 software (AB Sciex). The ESI source operation parameters were ion source, turbo spray; source temperature 500°C; ion spray voltage 5,500 V; ion source gas I, gas II, and curtain gas were set at 55, 60, and 25.0 psi, respectively; the collision gas was high. Instrument tuning and mass calibration were performed with 10 and 100 *μ*mol.l^−1^ polypropylene glycol solutions in QQQ and LIT modes, respectively. Based on the self-built database MetWare Database (http://www.metware.cn/) and metabolite information in public database, the materials were qualitatively analyzed according to the secondary spectrum information and the isotope signal was removed during the analysis. QQQ scans were acquired as multiple reaction monitoring (MRM) experiments with collision gas (nitrogen) set to 5 psi [23]. Declustering potential (DP) and collision energy (CE) for individual MRM transitions were done with further DP and CE optimization [22]. A specific set of MRM transitions was monitored for each period according to the metabolites eluted within that period.

### 2.5. Data Analysis

Statistical significance tests were performed to analyze the difference in MDA content between XL and D using the* t* test. All statistical analyses were conducted in R3.2 (R core). Analysis of variance (ANOVA) was conducted using accession, treatment, compound, and time point as factors, and the Tukey test (*p* ≤0.05) was used for mean comparison and separation. A hierarchical clustering heatmap was generated with log transformation of the mean value for each compound using the ‘pheatmap' package. An unsupervised principal component analysis (PCA) was selected to obtain the first understanding of the relationships among the data matrix. Then, a supervised orthogonal partial least-squares discriminate analysis (OPLS-DA) was performed using the ‘muma' package. The corresponding variable importance in projection (VIP) value was calculated from the OPLS-DA model. Differentially changed metabolites were identified when the VIP value was superior to 1. For pathway annotation, all the compounds were manually checked for their Kyoto Encyclopedia of Genes and Genomes name and number, thereafter, classified in component classes and putative pathways. Enrichment analysis was performed with the ‘clusterProfiler' package.

## 3. Results

### 3.1. Biochemical Responses of XL and D to Osmotic Stress

**Figure 1:**
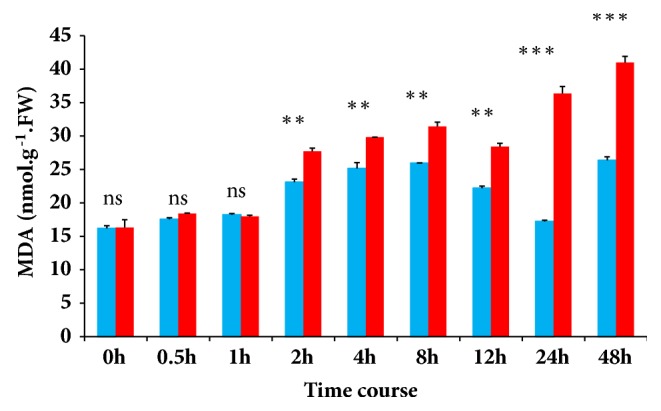
Quantification of leaf malondialdehyde (MDA) content. MDA was measured at 0, 0.5, 1, 2, 4, 8, 12, 24, and 48 h in the accession XL (blue bar) and D (red bar).* t* test comparison was performed between the two accessions. ^∗^Significant at* p* ≤ 0.05. ^∗∗^Significant at* p* ≤ 0.01. ^∗∗∗^Significant at* p* ≤ 0.001. ns, nonsignificant at* p* > 0.05.

Among 1,700 hulless barley accessions which were previously screened for tolerance to polyethylene glycol (PEG) 6000 simulated osmotic stress, XL and D were identified as the most tolerant and sensitive accessions, respectively. To further verify their osmotic stress tolerance, we evaluated the leaf malondialdehyde (MDA) content in the two accessions under osmotic stress. The results showed that both accessions experienced osmotic stress as the leaf MDA content increased gradually. However, the accession XL displayed significantly lower content of MDA in cells than the accession D (Figure 1). It is worth noting that, after 8 h, the leaf MDA content dropped significantly in XL, indicating a stout and adapted response to alleviate osmotic stress. Overall, these results confirmed that XL is highly tolerant and suffered less from osmotic stress than D.

### 3.2. An Overview of the Metabolites Detected in D and XL Leaves

**Figure 2:**
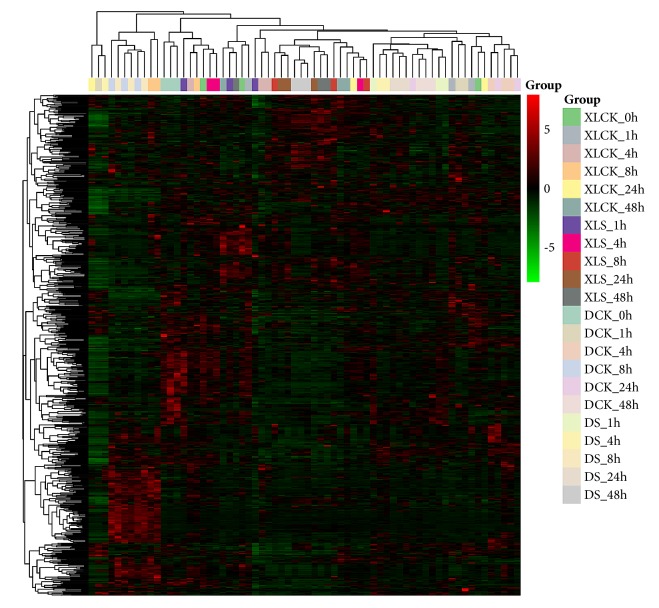
Heatmap hierarchical clustering of detected metabolite pools. Hierarchical trees were drawn based on detected metabolites in leaves of XL and D at 0, 1, 4, 8, 24, and 48 h in control (CK) and stress treatment (S). Columns correspond to accessions at different time points, while rows represent different metabolites.

In the present study, a total of 679 compounds were successfully detected and identified in leaves of XL and D during the different time points. The detected compounds could be grouped into 32 classes, predominantly, organic acids, flavone, nucleotide and its derivatives, and amino acid derivatives (Table 1). All metabolite data from leaves of both accessions in control and stress conditions were analyzed by hierarchical clustering to provide a global view of metabolite changes in the two treatments (Figure 2). No clear separation between D and XL could be observed in control treatment but XL and D were grouped into different clusters under osmotic stress. At some extent biological replicates were clustered together and there was no obvious separation between metabolomes from different time points. These results highlighted two principal features: (1) the importance of biological replicates and (2) the great difference in the metabolic reprogramming between D and XL under stress. The results of analysis of variance showed that, of the 679 measured metabolites, 395 (58%) were significantly and differentially accumulated between XL and D under osmotic stress. In addition, osmotic stress treatment significantly affected 513 (75%) metabolites mainly in the classes of flavonoids, nucleotide and its derivatives, organic acids, amino acid derivatives, and glycerophospholipids. Finally, the time points and accession by time point interaction also had significant effects on measured metabolites.

### 3.3. Differentially Changed Metabolites in XL and D under Time-Course Osmotic Stress

**Figure 3:**
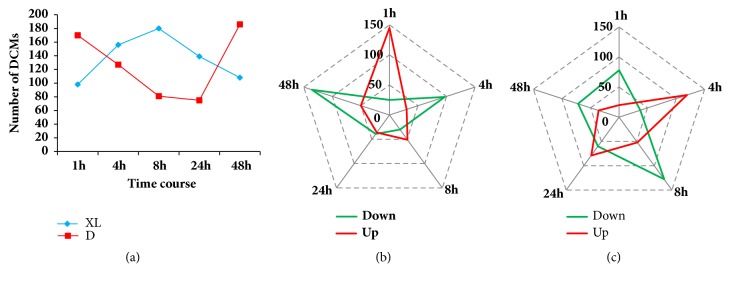
Temporal changes in metabolic reprogramming in XL and D under osmotic stress. (a) Differentially changed metabolites at 1, 4, 8, 24, and 48 h in XL (blue line) and D (red line) under osmotic stress. (b) Down- and upregulated metabolites in D at 1, 4, 8, 24, and 48 h under osmotic stress. (c) Down- and upregulated metabolites in XL at 1, 4, 8, 24, and 48 h under osmotic stress.

To determine the response of each accession to osmotic stress, we compared measurements of detected metabolites in the stressed plants to those in the control plants at a given time point and identified the differentially changed metabolites (DCMs). This resulted in a total of 356 and 408 DCMs in D and XL, respectively, during the whole stress period. Both accessions shared a great number of regulated compounds (251) belonging to the classes of flavonoids, glycerophospholipids, and amino acid derivatives which represent the core metabolome responsive to osmotic stress regardless of the tolerance levels (Supplementary Table S1). The two accessions conspicuously exhibited contrasting metabolic reprogramming under progressive stress. Upon exposure to stress (1 h), D sharply responded by up-accumulating numerous flavonoids and glycerophospholipids compounds (Figures 3(a) and 3(b)). Thereafter, the number of DCMs gradually decreased until 24 h, showing a weak ability of D to cope with prolonged stress. At 48 h, D was likely overwhelmed by the stress and thus strongly repressed hundreds of metabolites particularly, flavonoid compounds, which were found initially responsive to the osmotic stress. The top 20 DCMs during the different time points in D are summarized in Supplementary Figure S1.

In contrast to D, the number of DCMs, remarkably up-accumulated metabolites, increased under stress in the tolerant accession XL, suggesting a robust and lasting protective reaction (Figures 3(a) and 3(c)). The strongest metabolic responses in XL were observed at 4 and 8 h with many upregulated DCMs being flavonoid and glycerophospholipid compounds. Supplementary Figure S2 shows the top regulated metabolites during stress treatment in XL.

By comparing the temporal change in the metabolic responses of XL and D, we observed a huge difference in regulated metabolites at 8 h, demonstrating that 8 h is a critical time point for stress endurance in the tested accessions (Figure 3(a)). Therefore, DCMs detected at 8 h were in-depth scrutinized. Results revealed that XL strongly upregulated several metabolites (adenosine 3′-monophosphate, iP7G, adenosine 5′-monophosphate, uridine 5′-diphospho-D-glucose, A-nicotinamide mononucleotide, uridine 5′-diphosphate, and 2′-deoxyadenosine-5′-monophosphate) related to the class of nucleotide and its derivatives (pyrimidine and purine metabolism pathway), which were not observed in the sensitive accession. This implies that the up-accumulation of important metabolites from the pyrimidine and purine metabolism pathway is a key strategy for osmotic stress tolerance in hulless barley.

### 3.4. Alteration of Metabolites between XL and D under Progressive Osmotic Stress

**Figure 4:**
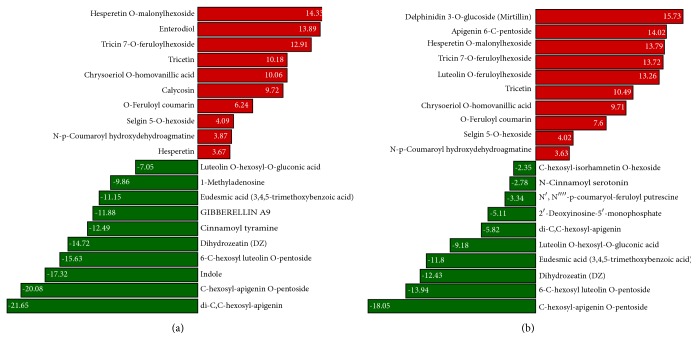
Overview of top regulated metabolites between XL and D under short-term osmotic stress. (a) Top 20 down- and up-accumulated metabolites between XL and D at 1 h. (b) Top 20 down- and up- accumulated metabolites between XL and D at 4 h.

To further understand the difference in metabolic reprogramming leading to contrasting osmotic stress tolerance in the two accessions, we compared the levels of each metabolite in D stressed plants to those in XL stressed plants (D_vs_XL). This led to the identification of DCMs at each time point and thereby, we performed the Kyoto Encyclopedia of Genes and Genomes (KEGG) pathway enrichment analysis. It is worth mentioning that, during the whole stress period, KEGG classifications “metabolic pathways” (ko01100) and “biosynthesis of secondary metabolites” (ko01110) were constantly the two most represented pathways. This was expected since these general pathways overlap with specific pathway classification and thus showed high numbers of DCMs. Accordingly, we mainly focused on specific pathways to uncover differences between the two accessions. At the onset of the stress, 118 and 167 DCMs were identified at 1 and 4 h, respectively, which were mostly up-accumulated metabolites enriched in KEGG pathways of flavonoid biosynthesis and phenylpropanoids. This highlighted the importance of these compounds in the early defense against harmful effects of osmotic stress in XL (Supplementary Tables S2, S3; Supplementary Figures S3, S4). The top 20 down- and up-accumulated DCMs presented in Figures 4(a) and 4(b) showed mostly similar metabolites between 1 and 4 h and a few time-specific DCMs.

**Figure 5:**
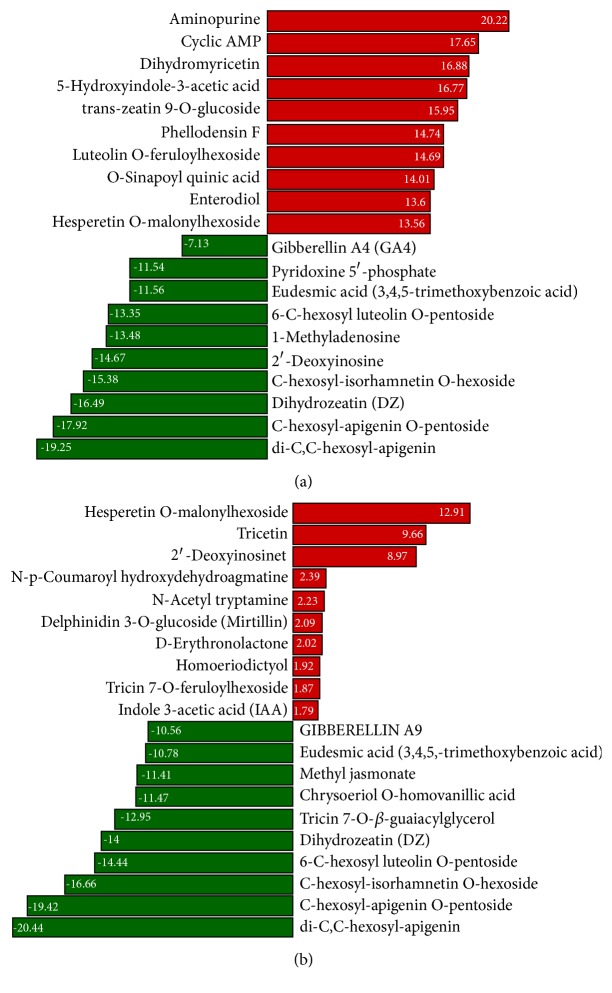
Overview of top regulated metabolites between XL and D under mid-term osmotic stress. (a) Top 20 down- and up-accumulated metabolites between XL and D at 8 h. (b) Top 20 down- and up-accumulated metabolites between XL and D at 24 h.

**Figure 6:**
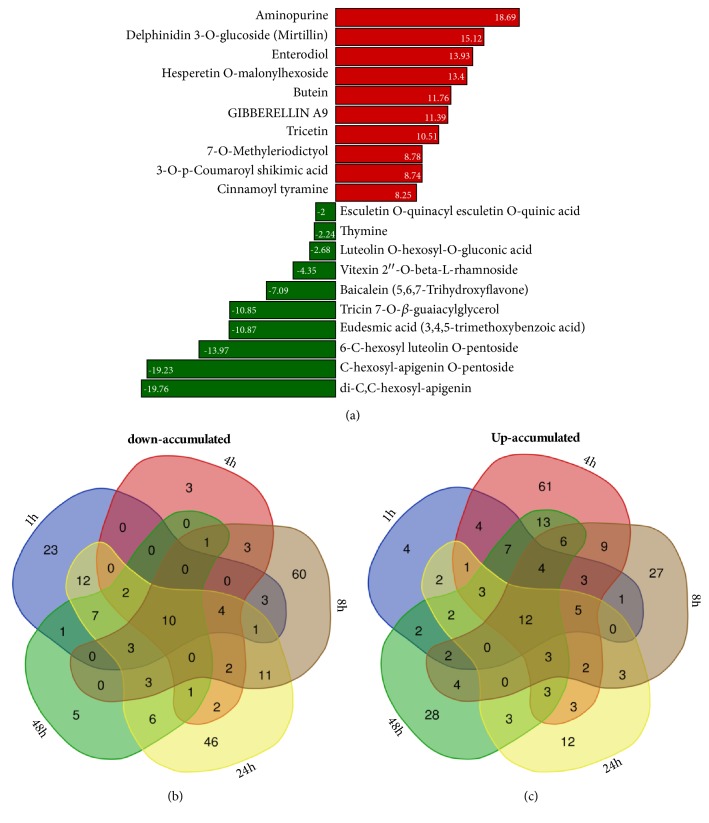
Overview of top regulated metabolites between XL and D under long-term osmotic stress. (a) Top 20 down- and up-accumulated metabolites between XL and D at 48 h. (b) Venn diagram representing the distribution of shared and common down-accumulated metabolites at different time points between XL and D. (c) Venn diagram representing the distribution of shared and common up-accumulated metabolites at different time points between XL and D.

In contrast to the early stages, major changes in DCMs were noted between XL and D at 8 h under stress. Metabolites from the classes of pyrimidine metabolism, purine metabolism, zeatin biosynthesis, and plant hormone signal transduction pathways were the most represented DCMs (Supplementary Table S4; Supplementary Figure S5). Importantly, the top down- and up-accumulated DCMs were quite different from those identified at the previous hours (Figure 5(a)), suggesting that the intensity of the stress at 8 h induced specific metabolic reprogramming which is different from the early metabolic responses. At 24 h under stress, we identified 164 DCMs including 110 down- and 54 up-accumulated compounds. KEGG pathway enrichment analysis revealed that phenylpropanoid biosynthesis, plant hormone signal transduction, and glutathione metabolism related metabolites were the most significantly changed between the two accessions at 24 h. Compared with the previous hour, most of up-accumulated metabolites were specific to 24 h, indicating a distinct metabolic reconfiguration (Figure 5(b); Supplementary Table S5; Supplementary Figure S6). Likewise, readjustment of metabolome between XL and D from 24 h to 48 h was obviously different. 132 DCMs including 92 newly up-accumulated compounds principally from the flavonoids class were recorded and represent the active compounds helping to mitigate effects of prolonged stress in XL (Figure 6(a); Supplementary Table S6; Supplementary Figure S7).

As shown in Figures 6(b) and 6(c), few metabolites were constitutively down-accumulated (methyl jasmonate, C-hexosyl-apigenin O-pentosid, gentisic acid, 4-hydroxy-7-methoxycoumarin-beta-rhamnoside, 2,3-dihydroxybenzoic acid, afzelechin, 6-C-hexosyl luteolin O-pentoside, luteolin O-hexosyl-O-gluconic acid, eudesmic acid, and di-C,C-hexosyl-apigenin) or up-accumulated (hesperetin 5-O-glucoside, hesperetin O-malonylhexoside, N-feruloyl tryptamine, tricin 7-O-feruloylhexoside, N-p-coumaroyl hydroxydehydroagmatine, delphinidin 3-O-glucoside, isotrifolin, N-acetyl tryptamine, tricetin, selgin 5-O-hexoside, O-feruloyl coumarin, and spiraeoside) during the whole stress period in XL compared to D but a large number of DCMs were found to be time-specific (Supplementary Table S7). Altogether, our results indicated that XL dynamically adjusts the metabolome in a time-dependent manner.

## 4. Discussion

### 4.1. Hulless Barley Regulated a Diverse Set of Metabolites in response to Osmotic Stress

Metabolites are the end products of cellular regulatory processes, and their levels are regulated as biological system responses to environmental stresses including drought [24]. As a resilient crop grown in the drought-prone environment of the Qinghai-Tibet Plateau, we hypothesized that hulless barley can provide novel insights into stress tolerance mechanism at the metabolic level. The time-course metabolic profiling of tolerant and sensitive hulless barley accessions under osmotic stress is presented for the first time in this study. We identified in leaves of the two accessions 679 metabolites, of which 513 (76%) were significantly regulated under stress. The number of stress-responsive metabolites identified in hulless barley is strikingly higher than previous reports in soybean (266), rice (89), Lotus species (198), tobacco (116), and* Arabidopsis* (82) [16, 24–27]. Therefore, there may be a great number of unreported stress-responsive metabolites that hulless barley synthesizes to protect itself from drought or related abiotic stresses inducing osmotic stress. Similar works by Gechev et al. [28] showed that* Haberlea rhodopensis*, a resurrection species with extreme resistance to drought, also regulates several unique metabolites and unidentified compounds with powerful stress protective functions. Interestingly, the tolerant accession XL significantly regulated a larger variety of metabolites than the sensitive accession, which may promote the establishment of a robust system to cope with the stress [29]. This result was expected given that Liang et al. [12] also observed that their tolerant hulless barley genotype significantly regulated a higher number of genes than the sensitive one under drought stress.

### 4.2. The Core Metabolome Responsive to Osmotic Stress Highlighted Novel Active Compounds in Hulless Barley

Metabolites from the classes of flavonoids, glycerophospholipids, and amino acid derivatives constitute the core metabolome responsive to osmotic stress in hulless barley. Interestingly, several KEGG pathways enriched in this core metabolome were also present in the top pathways reported in the stress-responsive transcriptome of the hulless barley cultivar Himalaya 10 [11]. Flavonoids are a group of multifunctional plant secondary metabolites that play a key role in protecting plants against abiotic stresses [30]. Drought leads to osmotic stress which causes an increase in reactive oxygen species (ROS) in plant cells resulting in extensive cellular damage and death [31]. The hydroxyl groups of flavonoids are able to mitigate effect of drought/osmotic stress through ROS scavenging [32]. Therefore, the strong accumulation of flavonoid compounds in hulless barley is a typical response to drought/osmotic stress as observed in several plant species such as* Arabidopsis*, broccoli, motherwort plant,* Scutellaria baicalensis*, etc. [32–34]. By investigating drought-responsive metabolites and their associated quantitative trait loci, Piasecka et al. [35] reported that most of the altered metabolites were flavonoids, particularly flavones glycosides, in 100 hulled barley genotypes. In this study, luteolin O-feruloylhexoside and dihydromyricetin were particularly highly accumulated in both accessions under stress; however, no previous studies have expressly linked these compounds to drought/osmotic stress response mechanisms in plants. Further in-depth investigations are needed to uncover the role of these novel stress-responsive metabolites in hulless barley and other plant species.

Glycerophospholipid metabolism is an important pathway that is significantly activated during stress [36]. Chmielewska et al. [37] observed many glycerophospholipids strongly accumulated under drought stress in hulled barley, indicating a conserved mechanism in hulled and hulless barley. Several glycerophospholipids were found highly accumulated in ryegrass,* Halogeton glomeratus, Hydrangea macrophylla*, in responses to different stresses such as drought, salt, aluminum toxicity, and chilling [38–40]. In the present investigation, phosphatidylcholines from the glycerophospholipid class were strongly induced particularly at the onset of osmotic stress. Phosphatidylcholines are a major source of choline for glycine betaine synthesis whose accumulation is an adaptive response to stress, intimately involved in the cellular ionic- and osmoregulation [31]. Giddings and Hanson [41] also underscored the implication of phosphatidylcholines in hulled barley response to drought stress, suggesting that the identification and proper manipulation of phosphatidylcholine encoded genes can lead to the genetic enhancement of stress tolerance in hulled barley.

Another important class of metabolites preponderant in the stress-responsive core metabolome of hulless barley was amino acids. Accumulation of amino acids is proposed to aid stress tolerance in plants, through osmotic adjustment, detoxification of ROS, and intracellular pH regulation [42]. Urano et al. [16] revealed that drought firmly induces the accumulation of amino acids in* Arabidopsis* leaves. Likewise, the most pronounced changes in metabolites under drought were observed in the amino acid levels, of which approximately half was significantly increased in various wheat cultivars [43]. In the case of the hulled barley, several authors reported that major osmotic/drought-responsive metabolites were amino acids, suggesting that hulled and hulless barley shared this common metabolic response feature [37, 44, 45]. Proline is a classical amino acid accumulated in various plants in response to a wide range of abiotic stresses [46, 47]. For example, in earlier studies, substantial increases of proline in hulled barley genotypes in response to water deficit were observed [45, 48]. It was proposed that proline could be regarded as a candidate stress tolerance biomarker in hulled barley [49]. But in this study, we found that changes in proline content were not very striking in both accessions under stress, suggesting that proline is not a prominent marker for osmotic stress response in hulless barley at least during the early stage. Our conclusion is in agreement with a previous report from Silvente et al. [42] who observed that proline content did not significantly change in soybean leaves under drought stress and referring to Fukutoku and Yamada [50], remarkable proline accumulation in soybean leaves occurred only when water stress becomes very severe. The most important amino acids significantly and strongly accumulated at different time points under stress revealed by our metabolic analysis were aspartic acid di-O-glucoside and phenylacetyl-L-glutamine. These compounds have been well described as involved in stress response in plants, including hulled barley [37, 51, 52].

### 4.3. XL Dynamically Readjusted the Metabolome in a Time-Dependent Manner to Sustain Osmotic Stress

Based on the variation observed in the expression levels of the same metabolites sampled at different time points under chilling stress in two rice cultivars, Zhao et al. [26] concluded that rice readjusts the metabolome in a time-dependent manner and emphasized that sampling at critical time(s) is essential for studying plant metabolomic responses to abiotic stresses. Similarly, Kim et al. [53] revealed the differential temporal response of* Arabidopsis* to salt at the metabolic level. They showed that the methylation pathway and glycine betaine biosynthesis pathway are systematically coordinated as an initial response to salt stress while glycolysis and sucrose metabolisms were prominent in response to long term exposure to salt. These conclusions matched our results in hulless barley which also reconfigured the metabolome in a dynamic way according to the sampling time. The synthesis of specific metabolites was activated at each time point of the stress with a significant number of unique metabolites [54]. Additionally, these results revealed the existence of a turning point for osmotic stress endurance (8 h) at which XL changed from an initial stress response program (short term response) to a long term adaptation [53]. Pyrimidine and purine nucleotides are known to directly participate in nucleic acid synthesis, providing the ultimate energy source for the synthesis of carbohydrates, lipids, peptides, and secondary metabolites [55]. Through the strong accumulation of pyrimidine and purine metabolites at 8 h, we postulated that XL tends to provide the major building blocks essential for safeguarding nucleic acids and for the formulation of adapted metabolic response to sustain osmotic stress at the later stage. This may explain the significant decrease of malondialdehyde content in leaves of XL after 8 h as shown in Figure 1.

A total of 12 up- and 10 down-accumulated metabolites were constantly regulated during the whole stress period in the tolerant accession XL compared with the sensitive one. These compounds constitute potential components of stress tolerance in hulless barley independently of the stress duration. The discovery of the genes regulating these candidate stress tolerance metabolites will undoubtedly offer novel genomic resources for the improvement of drought/osmotic tolerance in hulless barley and in other plant species as well.

## 5. Conclusions

Osmotic stress altered metabolite accumulation and induced differential adjustment of the metabolome in two contrasting hulless barley accessions. Several compounds from the flavonoids, amino acid, and glycerophospholipid classes were decisive to cope with osmotic stress and were defined in this study as the stress-responsive core metabolome. Metabolic reprogramming in XL under stress was dynamic and specific to the stress duration. During the critical time under stress (8 h), the tolerant accession XL exclusively regulated important metabolites related to the nucleotide and its derivatives which is likely a key tolerance mechanism. In sum, this study provided for the first time a large repertoire of stress-responsive metabolites which can be regarded as functional tools towards understanding the underlying regulatory networks and improving osmotic stress/drought tolerance in hulless barley. Future functional studies focusing on the novel stress tolerance metabolites identified in this work may shed light on unreported stress tolerance mechanisms in plants.

## Figures and Tables

**Table 1:** Classification of the 679 detected metabolites in hulless barley accessions into major classes.

**Groups**	**Number of Compounds detected**	**Groups**	**Number of Compounds detected**
Phenolamides	31	Amino acid derivatives	57
Coumarins	14	Alkaloids	6
Quinate and its derivatives	20	Phytohormones	19
Carbohydrates	15	Glycerolipids	18
Flavone	58	Glycerophospholipids	35
Lipids fatty acids	19	Nucleotide and its derivatives	55
Flavone C-glycosides	43	Indole derivatives	9
Others	31	Amino acids	28
Flavonol	24	Anthocyanins	4
Nicotinic acid derivatives	4	Vitamins	17
Tryptamine derivatives	9	Benzoic acid derivatives	13
Organic acids	66	Terpenoids	4
Cholines	5	Catechin derivatives	3
Flavonolignan	10	Flavanone	19
Hydroxycinnamoyl derivatives	30	Alcohols and polyols	4
Pyridine derivatives	2	Isoflavone	7

## Data Availability

The metabolic dataset data used to support the findings of this study are available from the corresponding author upon request.

## Supplementary Materials

Supplementary Materials
** Supplementary Table S1:** list of metabolites composing the core metabolome responsive to osmotic stress in hulless barley.** Supplementary Table S2:** list of the differentially changed metabolites between XL and D at 1 h.** Supplementary Table S3:** list of the differentially changed metabolites between XL and D at 4 h.** Supplementary Table S4:** list of the differentially changed metabolites between XL and D at 8 h.** Supplementary Table S5:** list of the differentially changed metabolites between XL and D at 24 h.** Supplementary Table S6:** list of the differentially changed metabolites between XL and D at 48 h.** Supplementary Table S7:** shared and specific differentially changed metabolites between XL and D at the different time points.** Supplementary Figure S1:** the top 20 differentially changed metabolites during the different time points in D.** Supplementary Figure S2:** the top 20 differentially changed metabolites during the different time points in XL.** Supplementary Figure S3:** KEGG enrichment of differentially changed metabolites at 1 h between XL and D.** Supplementary Figure S4:** KEGG enrichment of differentially changed metabolites at 4 h between XL and D.** Supplementary Figure S5:** KEGG enrichment of differentially changed metabolites at 8 h between XL and D.** Supplementary Figure S6:** KEGG enrichment of differentially changed metabolites at 24 h between XL and D.** Supplementary Figure S7:** KEGG enrichment of differentially changed metabolites at 48 h between XL and D.
