# Supplementary material for: Time-Course Comparative Metabolite Profiling under Osmotic Stress in Tolerant and Sensitive Tibetan Hulless Barley
Source: Biomed Res Int. 2018 Dec 23;2018:9415409. doi: 10.1155/2018/9415409 (PMC6323448; doi:10.1155/2018/9415409)
Supplement: Supplementary Materials — Supplementary Table S1: list of metabolites composing the core metabolome responsive to osmotic stress in hulless barley. Supplementary Table S2: list of the differentially changed metabolites between XL and D at 1 h. Supplementary Table S3: list of the differentially changed metabolites between XL and D at 4 h. Supplementary Table S4: list of the differentially changed metabolites between XL and D at 8 h. Supplementary Table S5: list of the differentially changed metabolites between XL and D at 24 h. Supplementary Table S6: list of the differentially changed metabolites between XL and D at 48 h. Supplementary Table S7: shared and specific differentially changed metabolites between XL and D at the different time points. Supplementary Figure S1: the top 20 differentially changed metabolites during the different time points in D. Supplementary Figure S2: the top 20 differentially changed metabolites during the different time points in XL. Supplementary Figure S3: KEGG enrichment of differentially changed metabolites at 1 h between XL and D. Supplementary Figure S4: KEGG enrichment of differentially changed metabolites at 4 h between XL and D. Supplementary Figure S5: KEGG enrichment of differentially changed metabolites at 8 h between XL and D. Supplementary Figure S6: KEGG enrichment of differentially changed metabolites at 24 h between XL and D. Supplementary Figure S7: KEGG enrichment of differentially changed metabolites at 48 h between XL and D. [file 9415409.f1.zip › 9415409.f8.pdf]

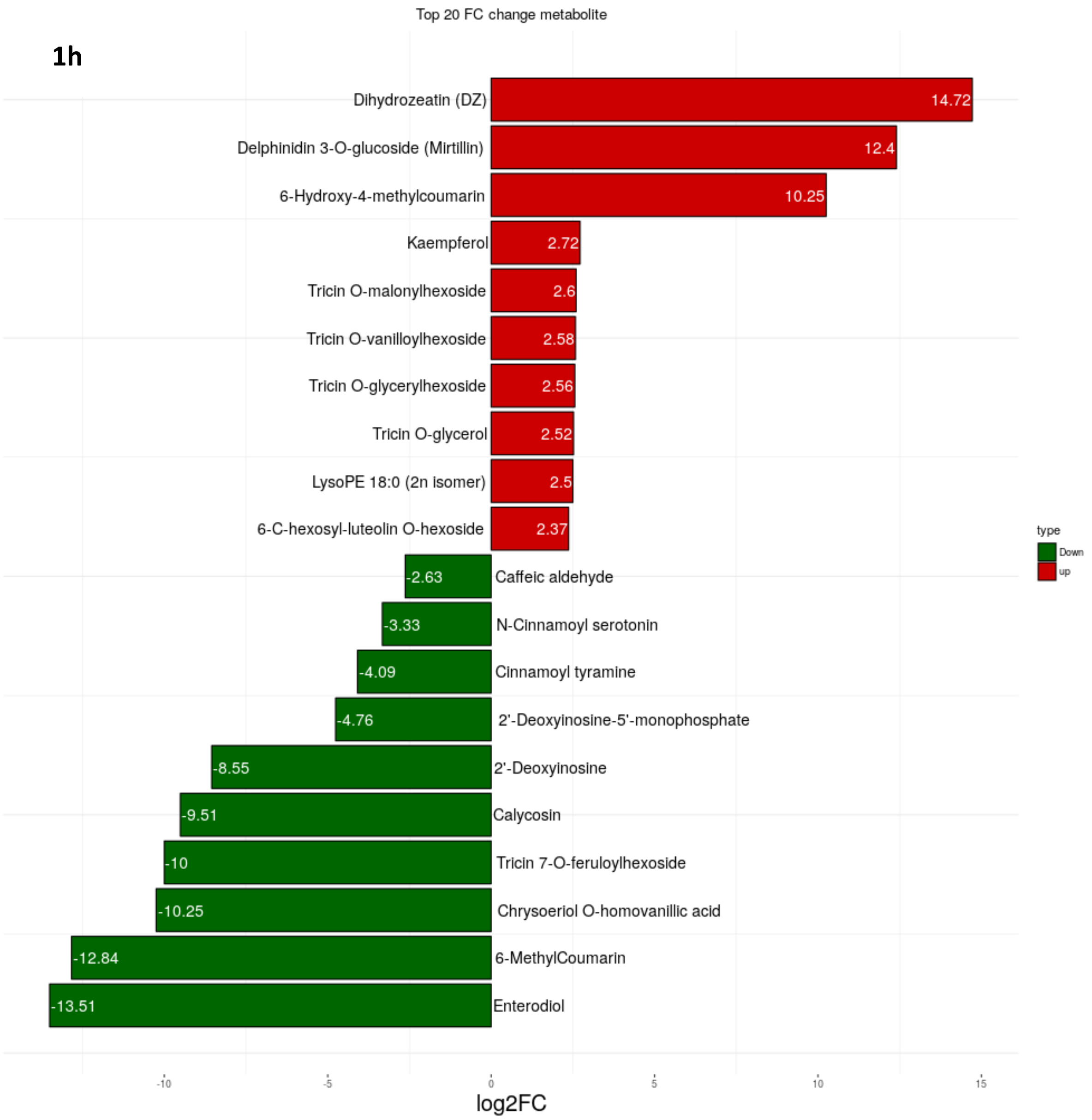

Supplementary Figure S1: The top 20 differentially changed metabolites during the different time points in D

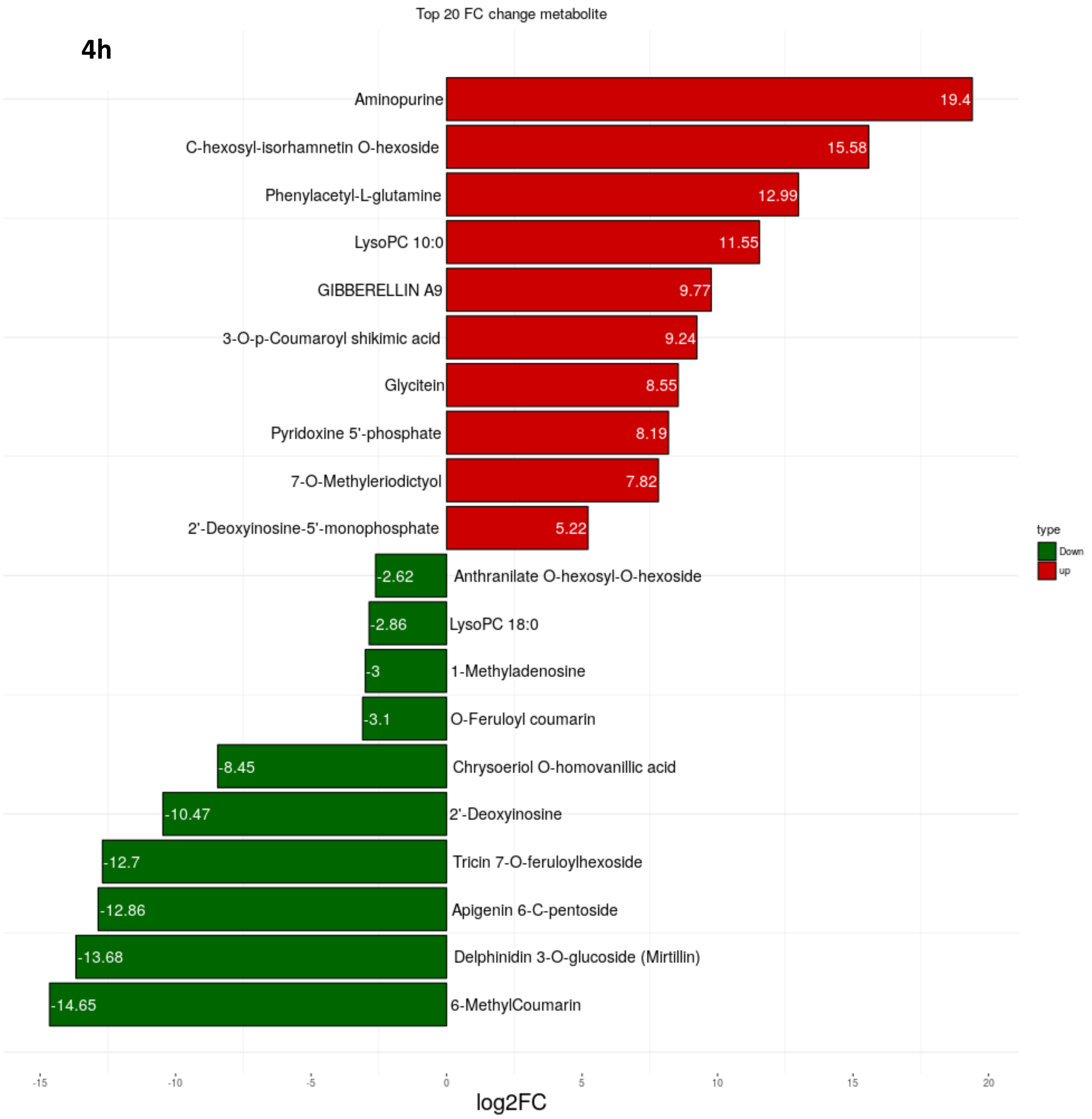

Supplementary Figure S1: The top 20 differentially changed metabolites during the different time points in D

8h

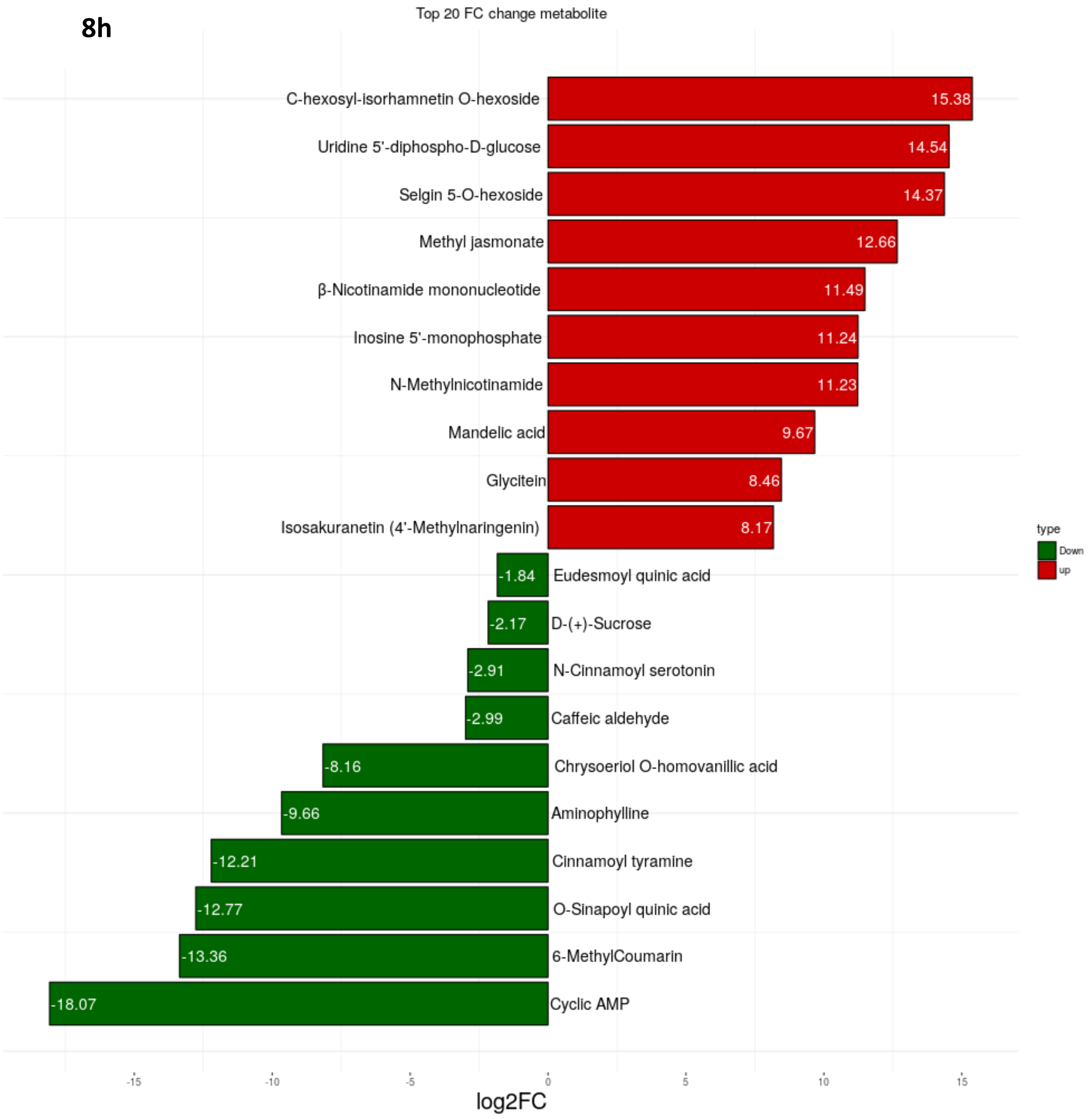

Supplementary Figure S1: The top 20 differentially changed metabolites during the different time points in D

24 h

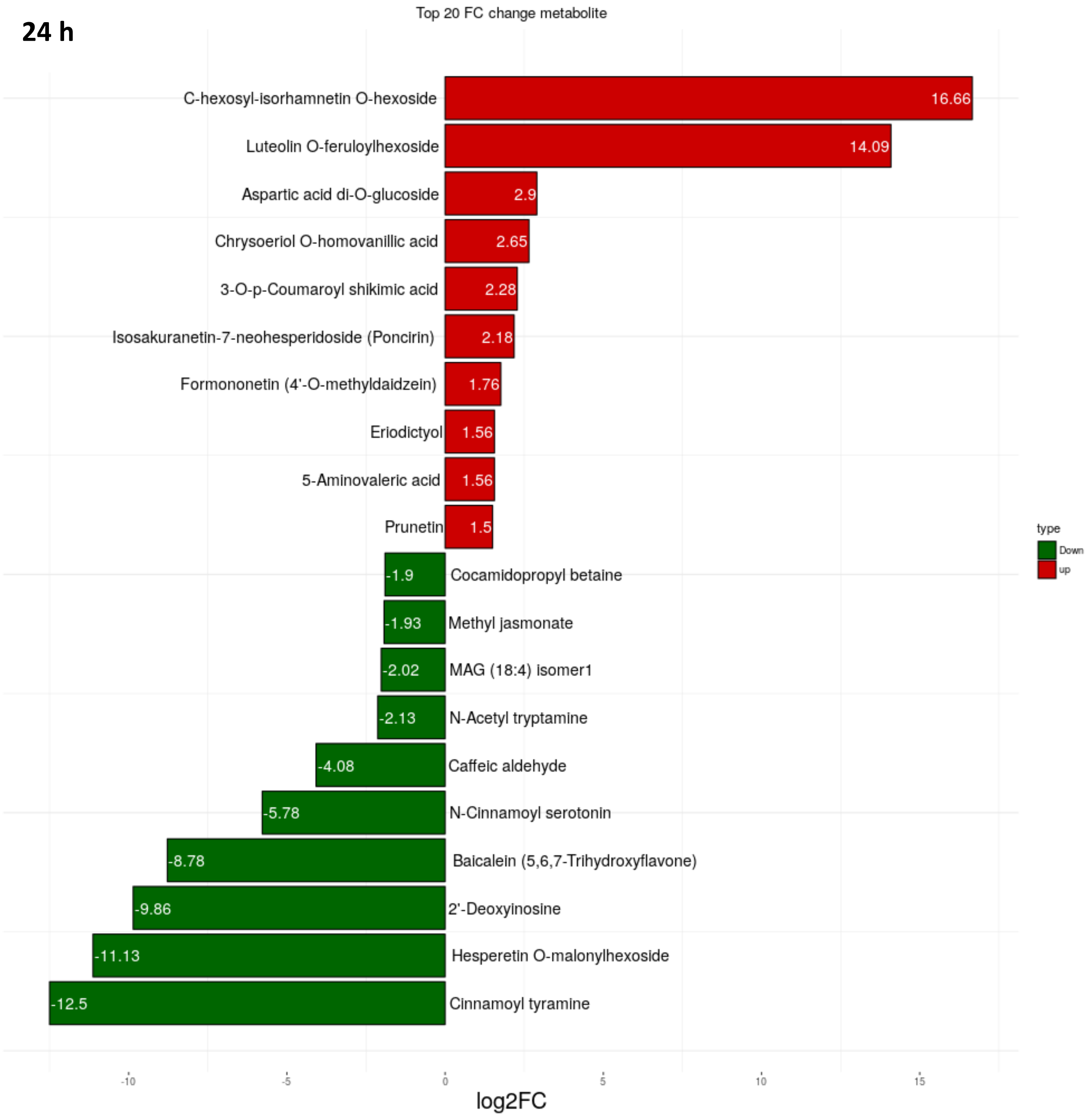

Supplementary Figure S1: The top 20 differentially changed metabolites during the different time points in D

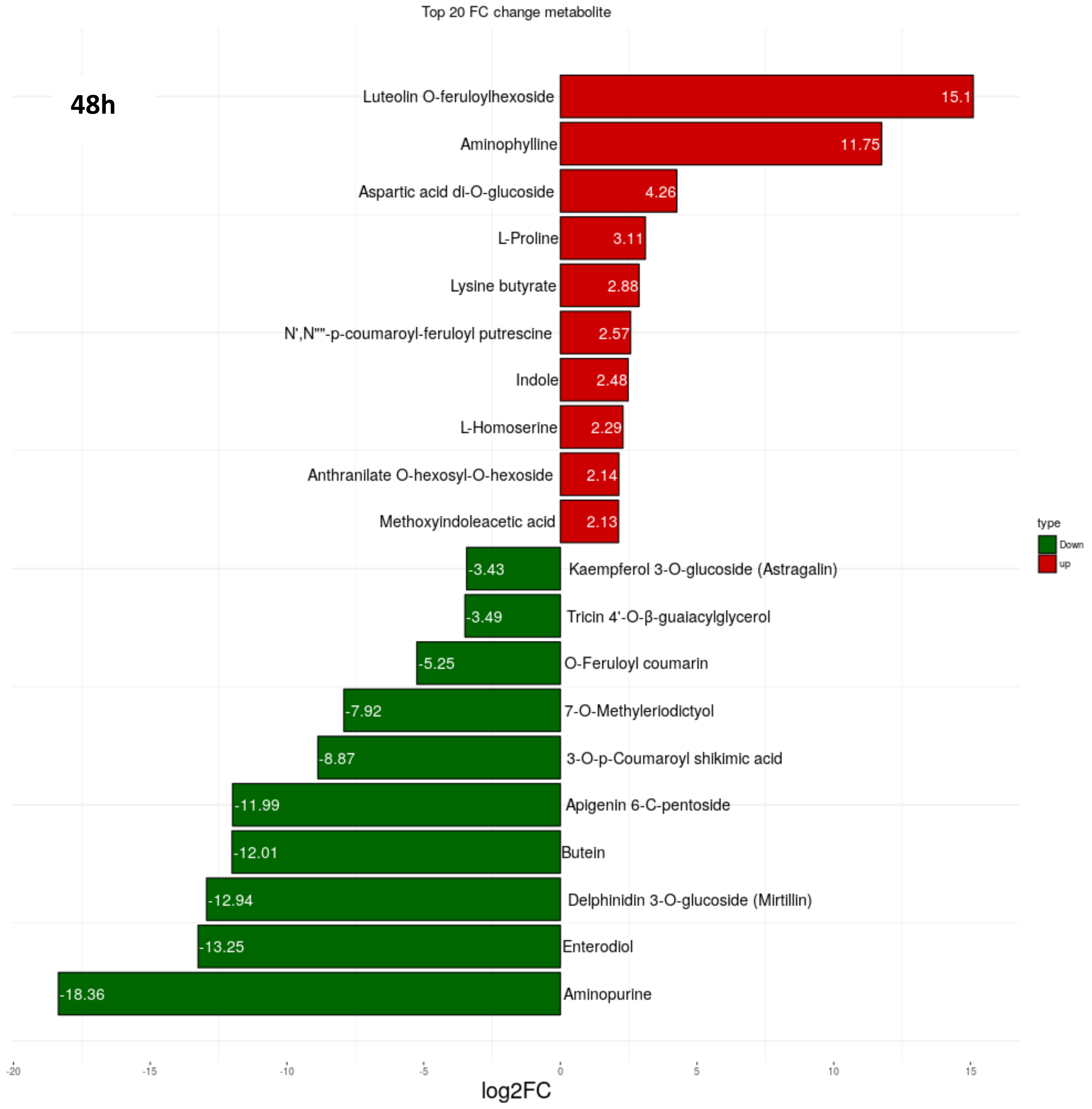

Supplementary Figure S1: The top 20 differentially changed metabolites during the different time points in D

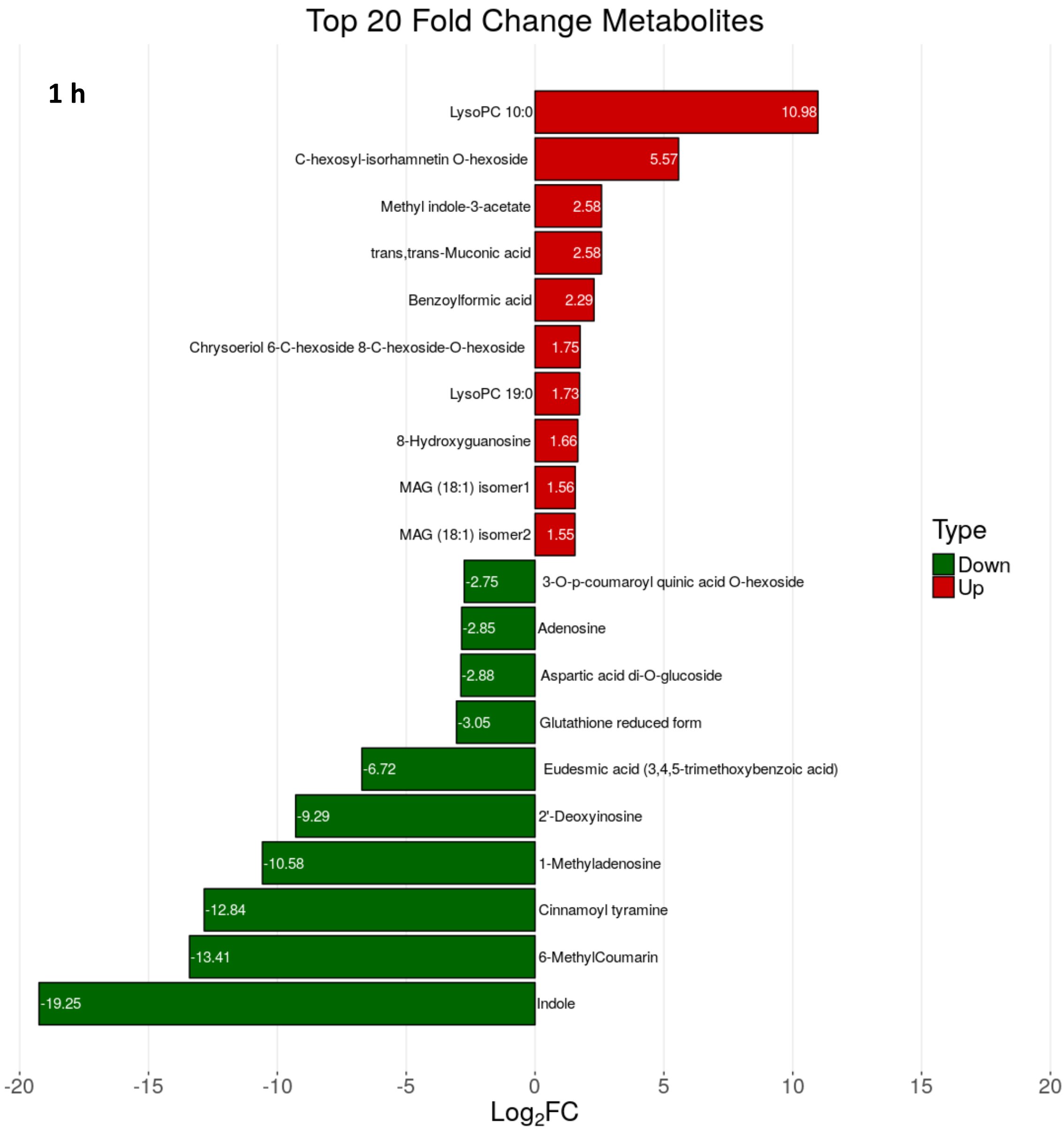

Supplementary Figure S2: The top 20 differentially changed metabolites during the different time points in XL

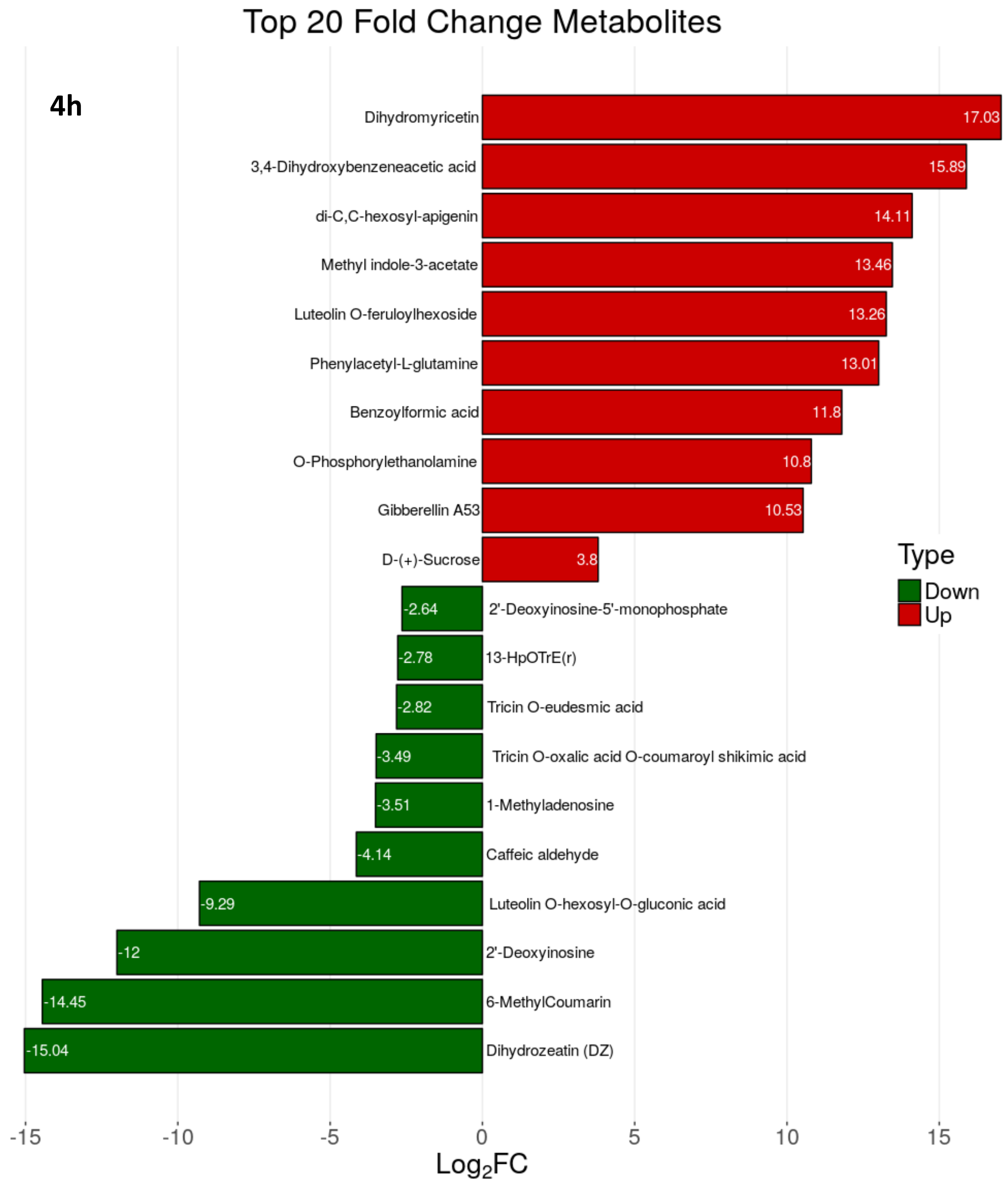

Supplementary Figure S2: The top 20 differentially changed metabolites during the different time points in XL

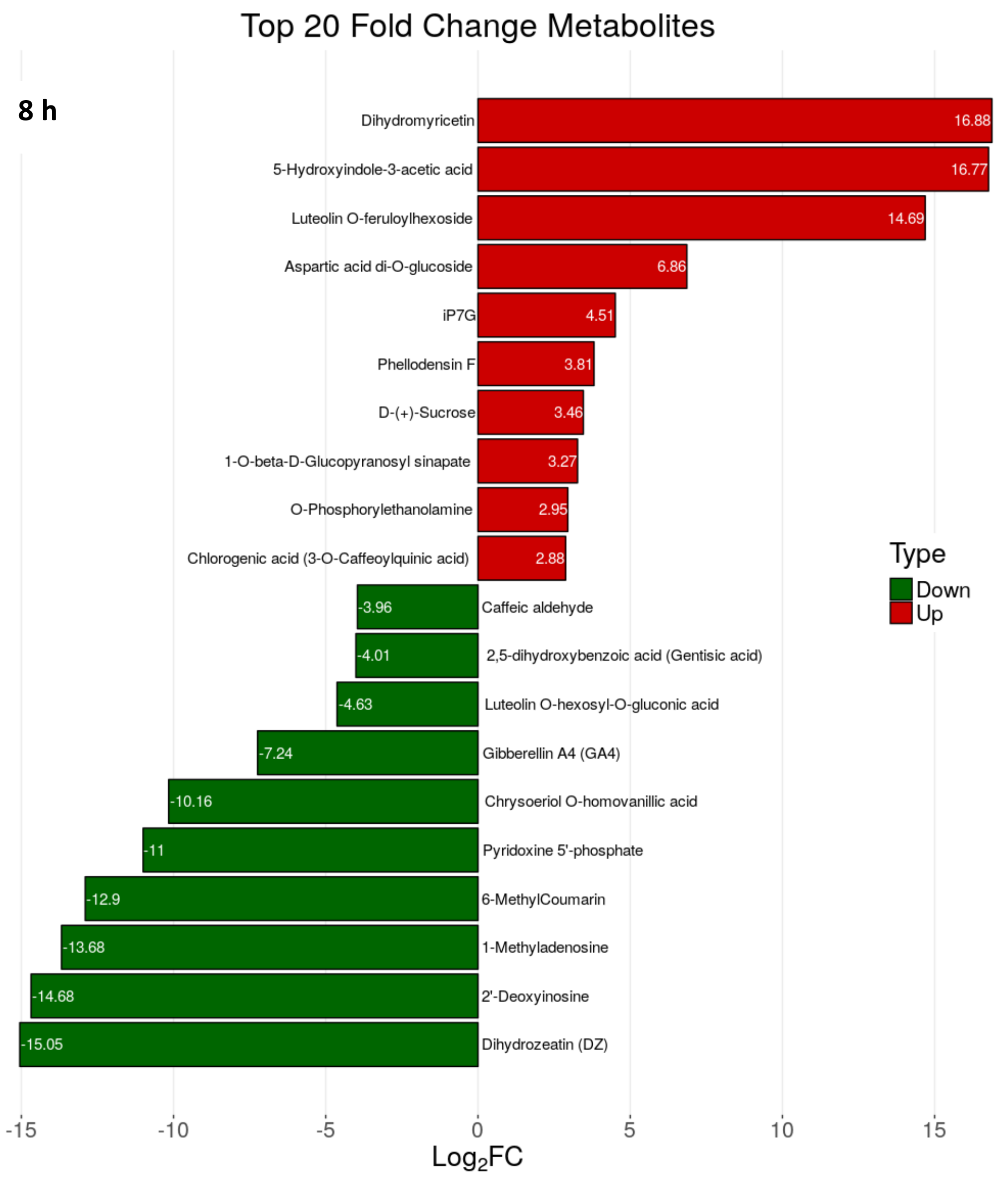

Supplementary Figure S2: The top 20 differentially changed metabolites during the different time points in XL

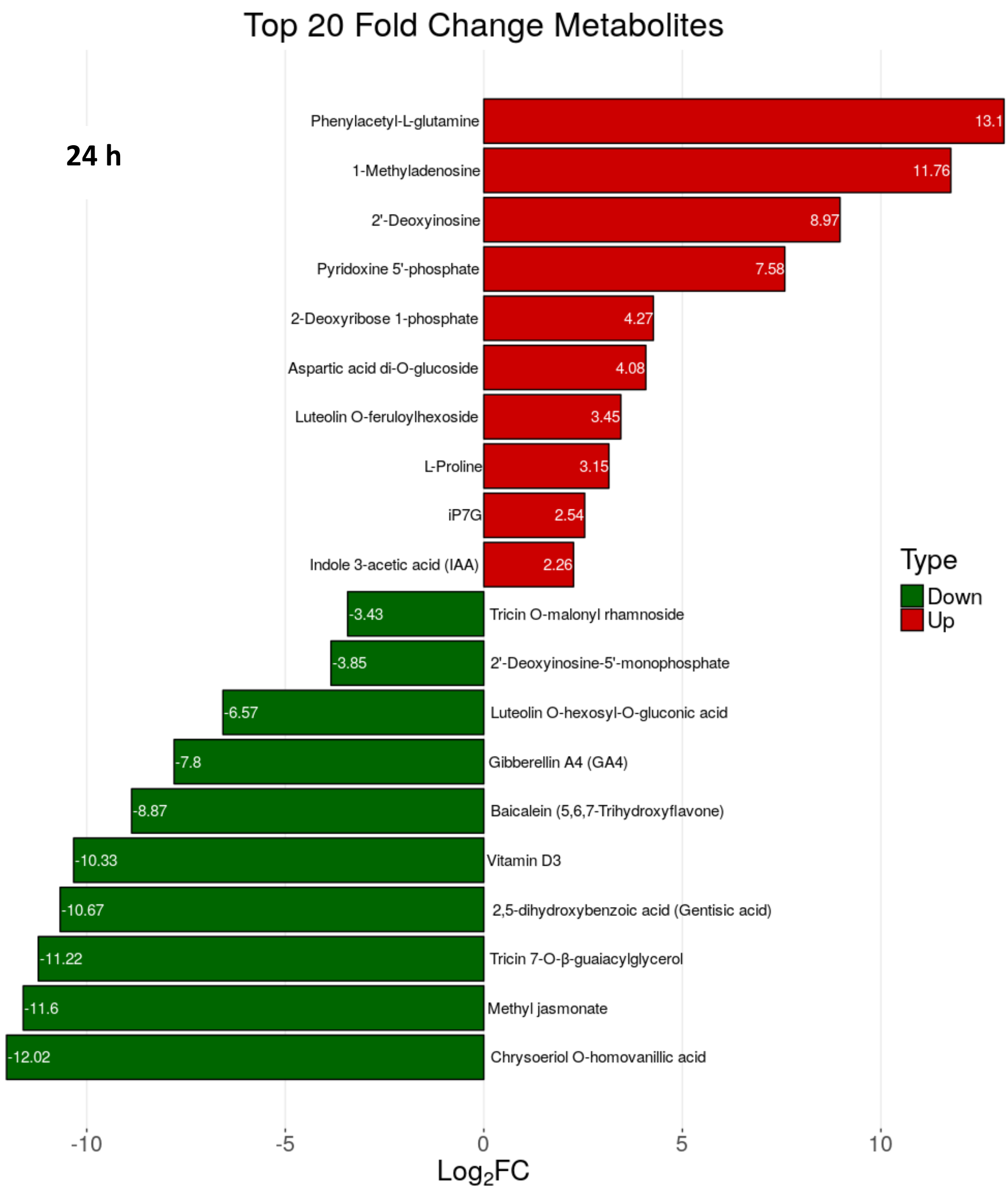

Supplementary Figure S2: The top 20 differentially changed metabolites during the different time points in XL

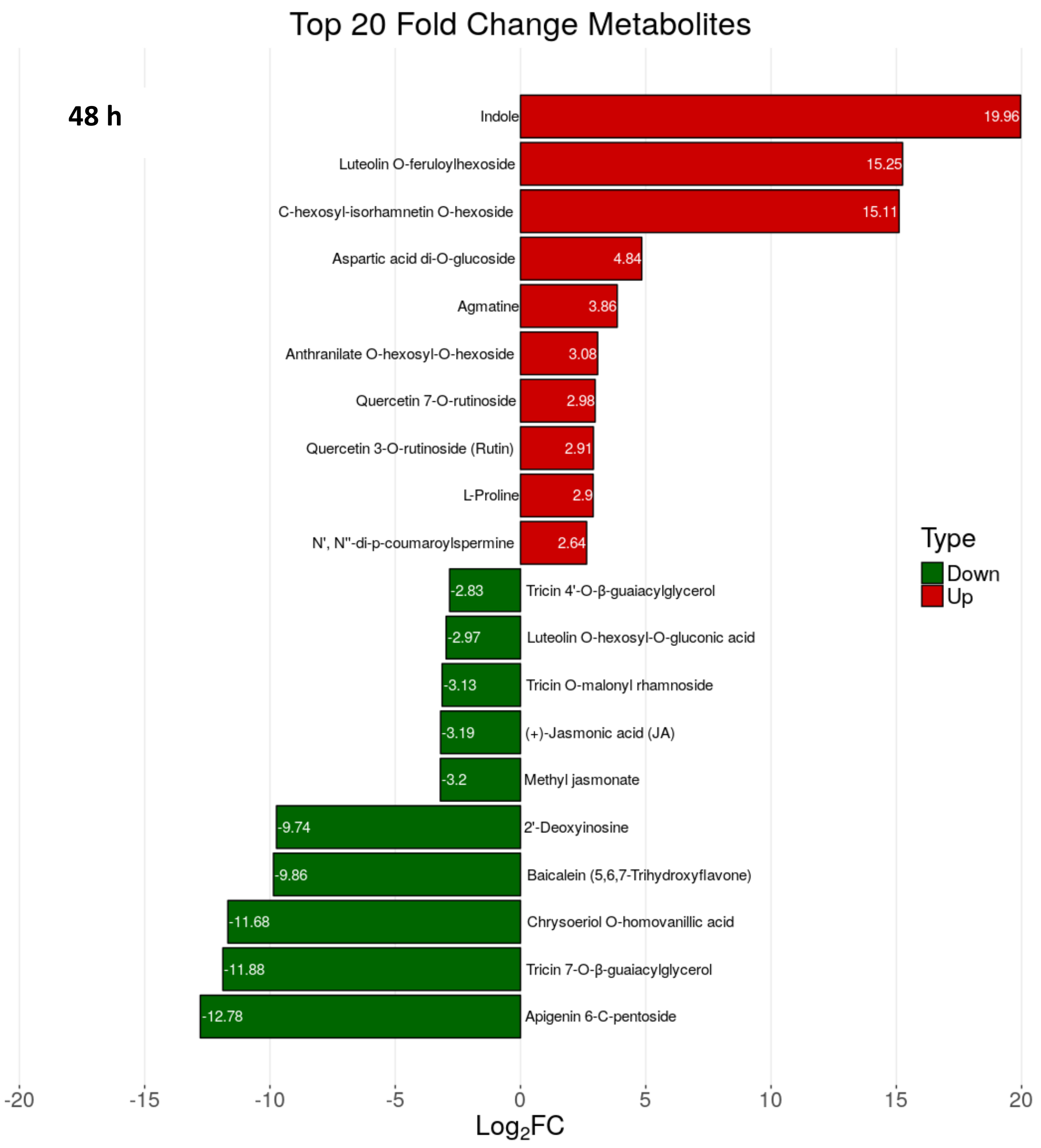

Supplementary Figure S2: The top 20 differentially changed metabolites during the different time points in XL

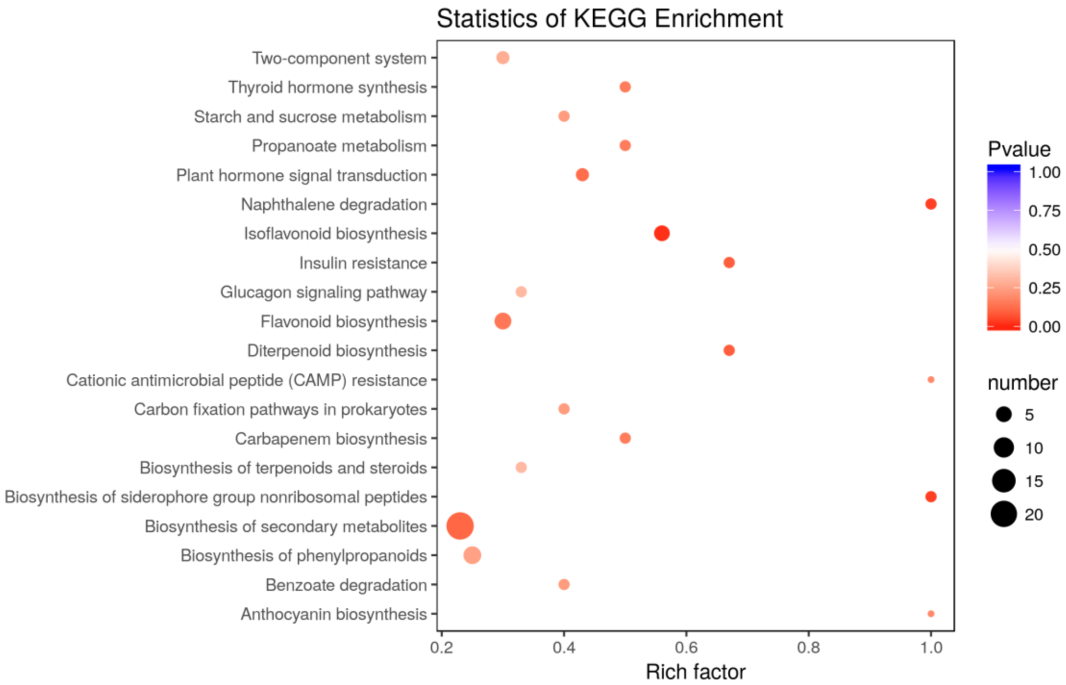

Supplementary Figure S3: KEGG enrichment of differentially changed metabolites at 1 h between XL and D

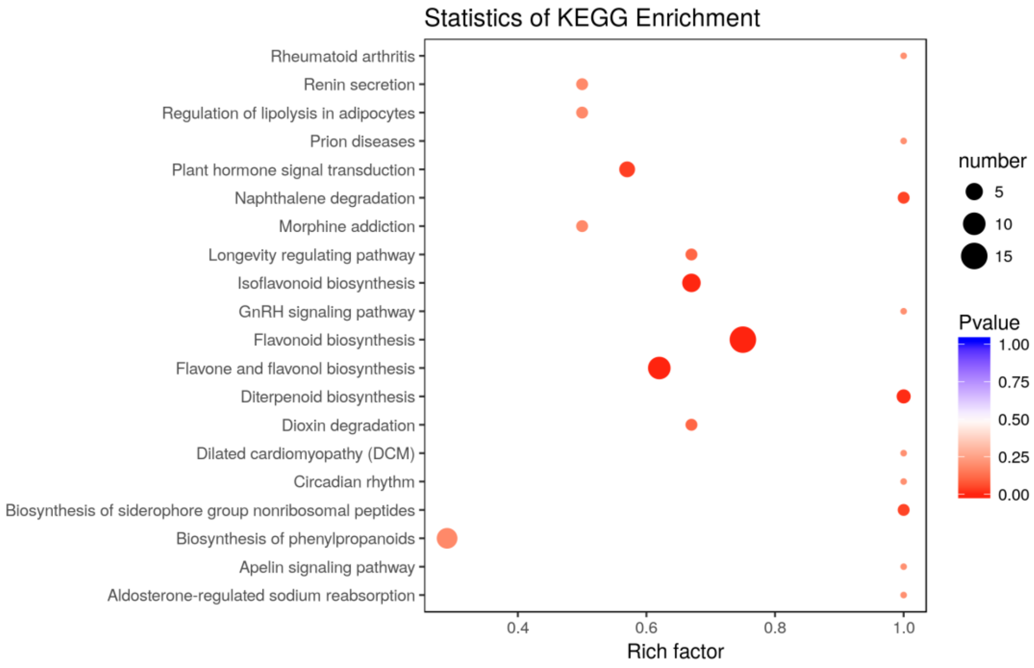

Supplementary Figure S4: KEGG enrichment of differentially changed metabolites at 4 h between XL and D

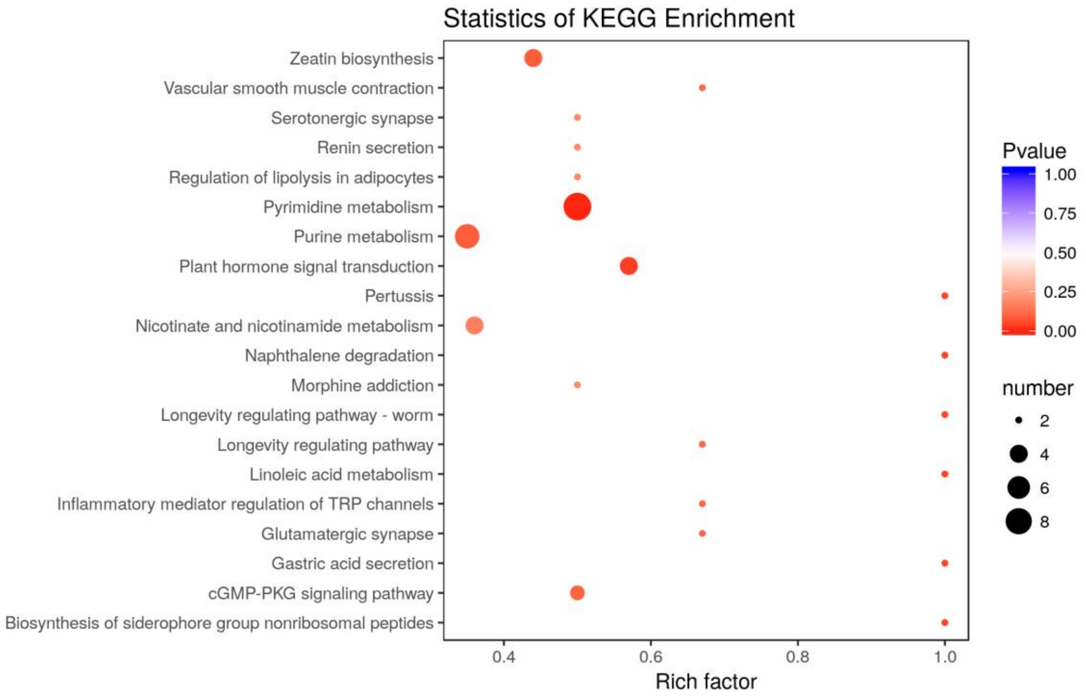

Supplementary Figure S5: KEGG enrichment of differentially changed metabolites at 8 h between XL and D

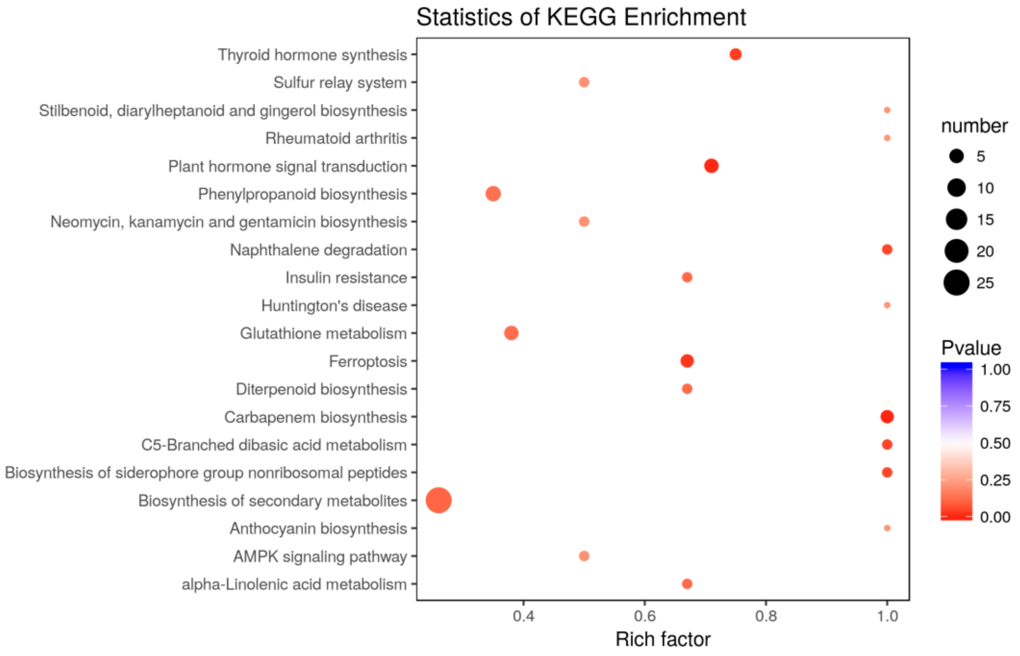

Supplementary Figure S6: KEGG enrichment of differentially changed metabolites at 24 h between XL and D

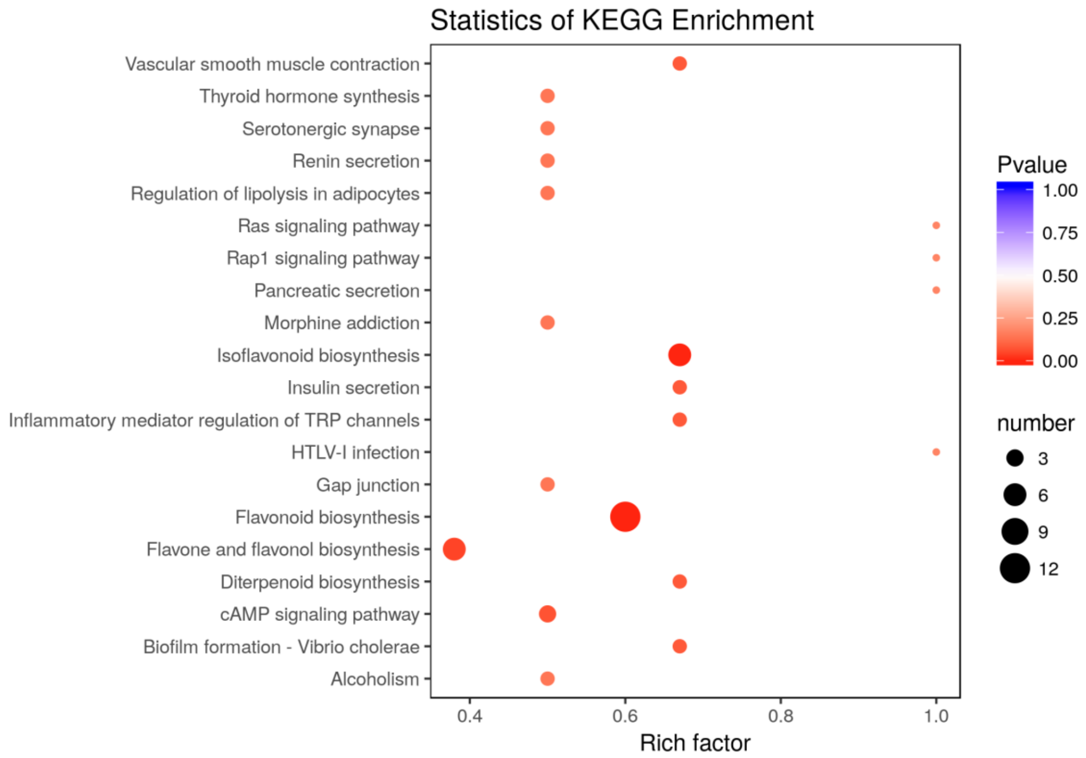

Supplementary Figure S7: KEGG enrichment of differentially changed metabolites at 48 h between XL and D
